# Assessing Soil Quality, Wheat Crop Yield, and Water Productivity under Condition of Deficit Irrigation

**DOI:** 10.3390/plants13111462

**Published:** 2024-05-24

**Authors:** Mohamed Emran, Omar M. Ibrahim, Asal M. Wali, Khaled M. Darwish, Rasha M. Badr Eldin, Maryam M. Alomran, Amira M. El-Tahan

**Affiliations:** 1 Arid Lands Cultivation Research Institute (ALCRI), City of Scientific Research and Technological Applications (SRTA-City), New Borg El-Arab City 21934, Egypt; memran@srtacity.sci.eg (M.E.); oibrahim@srtacity.sci.eg (O.M.I.); aeltahan@srtacity.sci.eg (A.M.E.-T.); 2 Department of Soil and Water Sciences, Faculty of Agriculture, Alexandria University, Alexandria 21568, Egypt; 3 Department of Biology, College of Science, Princess Nourah bint Abdulrahman University, P.O. Box 84428, Riyadh 11671, Saudi Arabia

**Keywords:** arid lands, calcareous soil, drought stress, silicon foliar application, supplemental irrigation, wheat production

## Abstract

Wheat is one of the most important cereal crops in Egypt and all over the world. Its productivity is adversely affected by drought due to deficient irrigation to provide nutrients required for plant growth. In a field experiment, silicon foliar applications at concentrations of 0, 200, and 400 mg L^−1^ were performed at different irrigation rates ranging from 1000 to 4000 m^3^ ha^−1^ to assess water irrigation productivity and wheat crop yield in a calcareous soil under arid climate conditions. Increased irrigation rates led to a significant increase in soil nutrient dynamics, as well as in the number and weight of grains per spike, leaf area index, grain yield, straw yield, and biological yield, with the exception of the weight of 1000 grains. Spraying with sodium silicate had a significant impact on grain yield and harvest index but did not significantly impact the other traits. Furthermore, the interaction between irrigation and silicate application rates showed significance only for grain yield, the number of spikes/m^2^, and the harvest index. Applying three times irrigation could produce the highest nutrient retention, wheat yield, and water irrigation productivity. No significance was observed between 3000 m^3^ ha^−1^ and 4000 m^3^ ha^−1^ irrigation, indicating a saving of 25% of applied irrigation water. It can be concluded that applying irrigation at 3000 m^3^ ha^−1^ could be a supplemental irrigation strategy. High wheat grain yield can be achieved under deficit irrigation (3000 m^3^ ha^−1^) on the northwestern coast of Egypt with an arid climate by spraying crops with sodium silicate at a rate of 400 mg L^−1^.

## 1. Introduction

It is well known that Egypt is one of the top importers of basic crops, where 40% of its imports are grains, vegetable oils, and animal proteins due to the limited arable lands and water resources. Principally, it was reported as the top country for wheat imports in the world, with an expenditure of USD 2.69 million thousand in 2020, equivalent to 5.60% of the world’s wheat imports, despite its production having reached 9.2 million tons of wheat in 2020 [1].

Consequently, there is an immediate necessity for effective management of agricultural production in Egypt to decrease land degradation and limited resources and to take a step forward toward self-sufficiency in agricultural productivity. Wheat (*Triticum aestivum* L.), family Poaceae, is known as Bread wheat [2] and is considered an excellent energy source due to its high contents of starch and protein [3,4]. Wheat cultivation on a large scale is feasible, and its yield offers long-term food storage solutions. The growth of wheat occurs within diverse farming systems, with production management practices being influenced by a range of agro-climatic factors, including temperature, rainfall, day length, soil type, and topography, as well as biotic and socioeconomic factors [5]. However, changes in precipitation patterns, due to natural or anthropogenic effects, are triggering yield damage, particularly in arid areas [6,7]. Drought, as one of the most common abiotic stresses on plants in these areas, results in global yield losses of 20% for wheat [8,9].

In arid lands, soil drought-related effects such as soil salinity and alkalinity have been recently reported as the most effective land degradation indicators directly affecting plant production and crop yield in sustainable agriculture. In addition, scarcity of irrigation has been reported to deteriorate plant physiology and productivity, thus threatening food security and development goals [10,11]. Moreover, the plant immune system has become, under these circumstances of arid climate, inadequate in mitigating external or internal stresses. Recent studies have used foliar applications of antiperspirants such as chitosan, tryptophan, and potassium silicate to enhance plant resistance against both biotic and abiotic stresses [2,12]. This type of application for enhancing plant nutritional requirements and consequently improving plant productivity is encouraged to reach the Sustainable Development Strategy of Egypt Vision 2030, which uses modern agricultural management practices to achieve national food security. Silicon relieves various environmental stresses, such as deficit irrigation regimes, drought, and high fluctuations in temperatures and ultraviolet radiation, as well as chemical stresses, such as salt, heavy metals, and nutrient imbalance [13]. Silicon has been presented through various investigations [14]. These applications aim to stabilize drought stress responses in plants and regulate the activity of certain antioxidant enzymes. In this context, enhancing the relevant physiological aspects of plant growth appears to work in tandem with plants’ endogenous stress responses and consequently improve plant productivity. Sodium silicate has alleviated drought stress in the early growth stages of the wheat crop. Many researchers have reported that silicon promotes plant growth and yield under drought [15], salinity [16], water deficit, osmotic stress [17,18], and deficiency of sulfur and potassium [19], whereas many research findings describe the impact of silicon on cultivars with varying drought tolerance [20,21]. The variability in the observed silicon effects may be attributed to differences in plant species and genotype, as evidenced by research aimed at elucidating the impact of silicon on herbivory tolerance [22,23].

The aim of our research is to fill a critical gap in the simultaneous understanding of crop response to abiotic stress and soil conservation through the study of the silicon effect on the drought tolerance of wheat genotypes and the impact of drought on soil characteristics in arid regions. We seek to address this gap by investigating the potential of foliar silicon applications to alleviate drought stress and minimize irrigation requirements in wheat crops under arid conditions for soil conservation purposes. So, we aim to provide simple guidance for soil conservation by optimizing irrigation practices and enhancing crop productivity in water-limited environments.

## 2. Results

### 2.1. Soil Response to Irrigation Treatments

Tukey’s HSD test at α < 0.05 was employed to examine the significance of variability among the four irrigation treatments at the three sampling times. According to soil moisture and most soil chemical parameters (Table 1), the irrigation treatments showed a significant increase in soil moisture contents. However, no significant changes were observed in soil pH values, indicating that the applied irrigation treatments did not significantly affect the moderate-soil-alkalinity conditions. Moderate soil salinity was observed and varied significantly among the irrigation treatments, with the lowest values being found in the third month compared with other periods. The highest DOC/SOC values were seen throughout the three irrigation treatments, with the DOC values varying just slightly among the treatments. Furthermore, the molar ratios of C/P and N/P were used to examine the C, N, and P balance to evaluate the soil nutrient balance (Table 1). Additionally, the soil C content was not enough to achieve the stoichiometric balance of soil nutrients. Moreover, it can be assumed that in all treatments, these soils can be classified as N- and P-limited according to their very low contents of organic C, N, and P causing an imbalance in soil nutrient dynamics. Nevertheless, a substantial enhancement in nutrient dynamics was observed, demonstrating high statistical significance (*p* < 0.05), across the irrigation periods, with the highest values being observed in those treatments receiving 4000 m^3^ ha^−1^ compared with the other irrigation treatments. Therefore, we may assume that increasing the number of irrigation applications would result in enhanced soil nutrient cycling and consequently promote the stoichiometric balance of soil nutrients.

Furthermore, an increase in the amount carbon dioxide (CO_2_) released from the soil, referred to as its respiration capacity, was noted. The elevated CO_2_ emissions from these soils with respect to their low contents of soil carbon pools can be attributed to the imbalance in soil nutrients that in turn increased the soil carbon loss assessed as C-CO_2_. No significant variability was observed in the CO_2_ emitted from soil among the irrigation treatments at each sampling time, but high significance among the three sampling times was found for CO_2_ (F = 45.46, *p* < 0.001), C-CO_2_ (F = 45.46, *p* < 0.001), and C-CO_2_/SOC (F = 27.26, *p* < 0.001). This could imply that the significance of nutrient cycling found for molar C, N, and P did not contradict the carbon loss supplied as C/CO_2_, suggesting that nutritional cycling was essential despite the minor carbon loss. Accordingly, the nutrient contents varied significantly due to the changes in soil moisture regimes despite the fact that the applied irrigation rate was below the seasonal recommended water content of 5000 m^3^ ha^−1^ (Table 1).

Soluble soil cations and anions were measured (Table 2). Moreover, Tukey’s HSD test at α < 0.05 among the four irrigation treatments at the three sampling times showed highly significant variability in all soil cations but with no significant changes in soil anions.

The cation exchange capacity (CEC) and the effective percentages of cations are presented in Table 3. The CEC of the soil and the effective percentages of calcium (Ca _Effective_) and magnesium (Mg _Effective_) presented a significant increase (*p* < 0.05) with the increase in the irrigation rate but no significant changes were observed.

**Figure 1:**
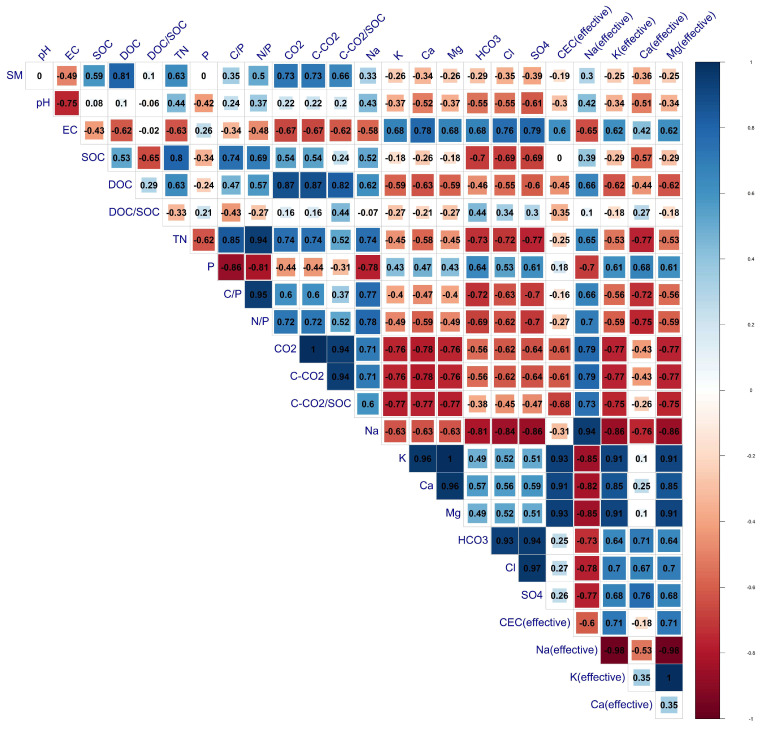
Pearson correlation matrix among all the studied soil parameters.

The correlation coefficients among all the studied soil parameters were measured (Figure 1). It can be found that soluble soil cations and anions decreased with the increase in soil organic reserve and nutrient limitation parameters. But the effective percentage of soil cations increased positively with the increase in contents of soil organic reserve parameters. Soil moisture was highly correlated with soil organic C and total N. Soil organic reserve parameters including SOC, TN, and DOC were positively correlated with the nutrient limitation parameters (C/P and N/P), soil moisture (SM), and soil carbon loss parameters (CO_2_ and C-CO_2_). Soil organic carbon was negatively correlated with DOC/SOC, indicating that increasing the irrigation rate may increase the transformation rate of dissolved or labile organic compounds into soil carbon pools (stable carbon forms). Additionally, soil carbon loss as a function of SOC (C-CO_2_/SOC) showed to be highly correlated with soil moisture and labile carbon fraction (DOC). This observation confirms that the dynamics of soil organic matter storage, as indicated by soil organics, exhibit nutrient-limiting increases with the increase in the irrigation rate.

### 2.2. Factor Analysis for Soil Response

Factor analysis was employed for all soil analyses (Table 4). The first three factors accounted for 85% of the total variance. The first factor, which explained 59% of the total variance, exhibited high positive loadings (>0.50) for electrical conductivity; soluble potassium, calcium, and magnesium; cation exchange capacity; and effective percentage of P and Mg. On the other hand, it displayed high negative loadings (>−0.50) for soil respiration (CO_2_), carbon loss as C-CO_2_, C-CO_2_/SOC, and soluble and effective sodium.

The structure of the second factor accounted for 17% of the total variance, displaying high positive loadings (>0.50) for soil pH, soil organic carbon, total nitrogen, and molar C/P and N/P ratios, as well as soluble and effective sodium. Conversely, high negative loadings (>−0.50) were observed for the dissolved proportion of SOC expressed as DOC/SOC, total P, bicarbonates, chlorine, SO_4_, and effective Ca. The third factor explained 9% of the total variance with high negative loadings (>−0.50) for soil moisture, soil organic carbon, dissolved organic carbon, total nitrogen, carbon loss, and C-CO_2_/SOC directly.

**Figure 2:**
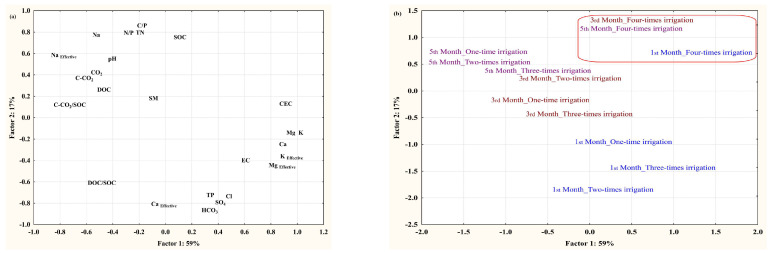
Relations between the first two factors: (**a**) factor loadings and (**b**) factor score values, red rectangle in (**b**) represents sampling times for the four-times irrigation.

The distribution of factor loadings in the relationship between the first-two-factor structures is shown in Figure 2a. Figure 2b also displays the distribution of factor scores in the relationship between the first two factors.

### 2.3. Wheat Yield Assessment

The main parameters for assessing the wheat crop yield (Table 5), showed that grain number/spike, grain weight/spike, and the number of spikes/m^2^, as well as the LAI of wheat, grain yield, biological yield, straw yield, and harvest index, were significantly increased by increasing the number of irrigation applications. For example, grain/spike increased from 32.93 under I1 to 44.97 under the I4 treatment. Also, grain weight/spike increased from 1.04 g to 1.53 g under the I1 and I4 treatments, respectively. The LAI increased from 1.58 to 2.19 under the I1 and I4 treatments, respectively. Nonetheless, no significant differences at *p* < 0.05 were observed in the weight of 1000 grains. Grain yield, biological yield, and straw yield showed the highest significant increases (*p* < 0.05) under the two times irrigation (I2) treatment because of the soil’s physical and chemical development at this irrigation rate. Under sodium silicate treatments, grain yield increased under the application of 200 mg L^−1^, but HI it increased under 400 mg L^−1^. The statistical interactions applied between irrigation and silicate treatments resulted in high significance only for spikes/m^2^, grain yield, and harvest index.

Five effect size statistics were used to assess the relative importance of the studied factors. They were eta squared ($\eta^{2}$), partial eta squared ($\eta_{p}^{2}$), omega squared ($\omega^{2}$), partial omega squared ($\omega_{p}^{2}$), and epsilon squared ($\varepsilon^{2}$).

### 2.4. Grain Yield Assessment

Among the wheat yield assessment parameters, grain yield, harvest index, and number of spikes/m^2^ were tested with Tukey’s HSD test (Table 6) to check their significant response to irrigation and silicate treatments and their interactions. Highly significant differences at *p* < 0.05 were observed among all applied treatments for the three parameters. The data summarized that the lowest values of grain yield (0.95-ton ha^−1^), harvest index (0.11), and number of spikes/m^2^ of the wheat crop (378.7) were for the interaction of one time irrigation with no Na-silicate. The four times irrigation treatment with 400 mg L^−1^ silicates (the highest application rate) yielded the highest values for both grain yield and harvest index. The highest values for the number of spikes were found under four times irrigation with no silicate. As the interaction between irrigation and silicate was significant for grain yield, harvest index, and number of spikes/m^2^, the response of these traits to irrigation depended on the silicate level, so high grain yield and harvest index were obtained under the four times irrigation treatment with 400 mg L^−1^ silicates.

**Figure 3:**
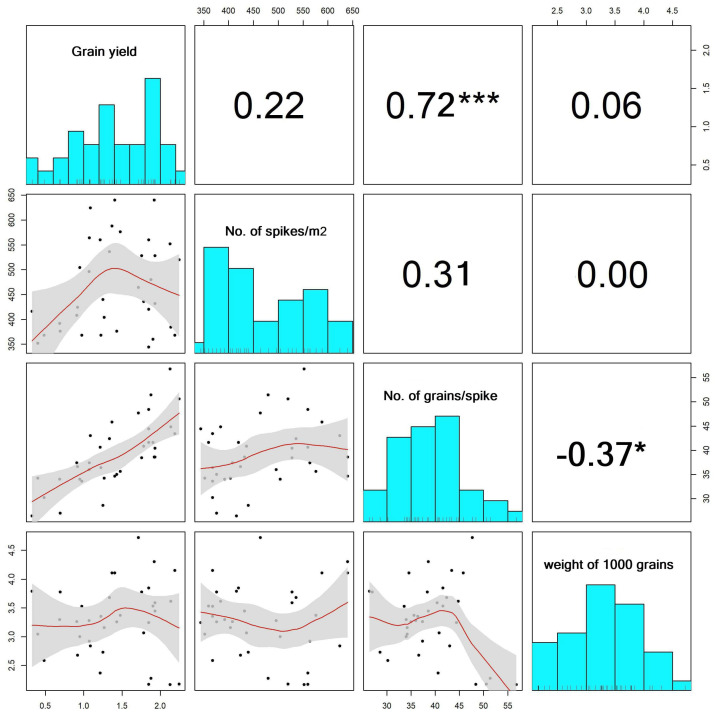
Correlation matrix among grain yield, number of spikes/m^2^, number of grains/spike, and weight of 1000 grains. ***, *p*-value < 0.001; *, *p*-value < 0.05; and NS, *p*-value > 0.05.

The correlation matrix depicted in Figure 3 illustrates the relationship between the number of spikes per square meter, the number of grains per spike, the weight of 1000 grains, and grain yield. The upper triangle represents the correlation coefficients of each pair of traits (parameters), while the lower triangle visualizes the trend relationship between the two variables. The diagonal histograms of the matrix show the distribution pattern of each trait. The matrix reveals that the traits were approximately normally distributed and that the number of grains/spikes was positively correlated with grain yield (r = 0.72, *p* < 0.01). The trend relationship of this correlation increased linearly along with the increase in the value of grain yield. A significant negative correlation was observed between the number of grains/spikes and the weight of 1000 grains (r = −0.37, *p* < 0.05). However, no significant correlation was observed for grain yield against the numbers of spikes/m^2^ (r = 0.22) and the weight of 1000 grains (0.06). The trend relationship between the number of spikes/m^2^ and grain yield was linear with a slight slope, which means a weak effect on grain yield. The trend relationship between the weight 1000 grains and grain yield formed a linear trend when the weight of 1000 grains increased by more than three grams.

**Figure 4:**
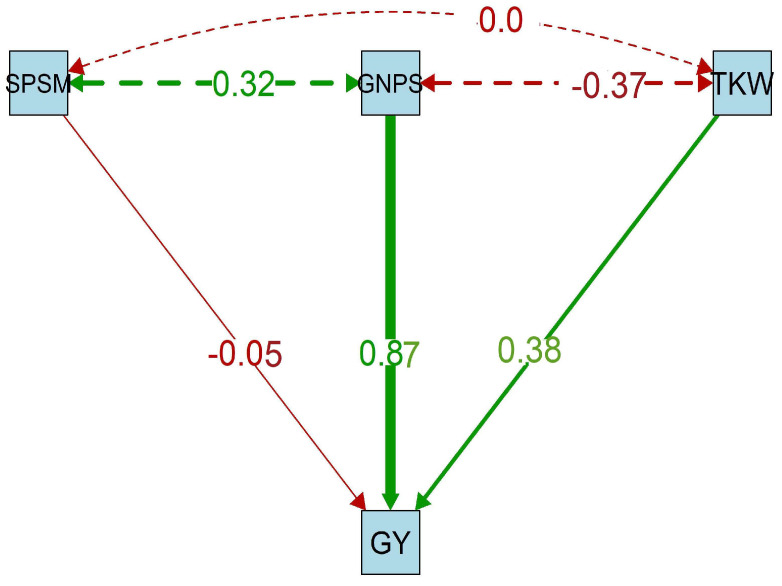
Path analysis of grain yield as affected by the number of spikes/m^2^, number of grains/spike, and weight of 1000 grains and their interaction effects. Green arrows indicated positive effect; red color indicates negative effect. Single headed arrows indicate direct effect, double headed arrows indicate correlation.

Figure 4 illustrates the path diagram resulting from path analysis, revealing the direct effect of yield components on grain yield and their correlations. The analysis identified the number of grains per spike (0.87) as the most influential and crucial trait affecting grain yield. The number of spikes/m^2^ was not adequate for grain yield, as its coefficient was (−0.05). Despite that, the number of grains/spike was positively correlated by the number of spikes/m^2^ (0.31), while it was negatively affected by the weight of 1000 grains (−0.37), as there was no effect between the last two parameters (0.00).

### 2.5. Grain Yield Reduction

Table 7 presents the relative yield reduction affected by the number of irrigation applications at the silicon application rates. The data show the highest reduction under the one time irrigation treatment (an average of 53.7%) compared with the four times irrigation treatment. The highest reduction was under zero-silicate application in this treatment (70.9%). The lowest reduction was 6.4% under three times irrigation compared with the four times irrigation treatment, which also saved about 25% of the amount of irrigation.

### 2.6. Irrigation Water Productivity under Applied Treatments

The irrigation water productivity values are expressed as kg m^−3^ and concern the applied irrigation water (AIW) under irrigation and sodium silicate treatments (Table 8). Regarding the averages presented by the two observed seasons, the highest value of IWP was 2.1 kg m^−3^ under the one time irrigation treatment, while the lowest was 1.09 kg m^−3^ under the four times irrigation treatment. Within the treatments, the highest IWP was 2.98 kg m^−3^ under the one time irrigation treatment at the 400 mg L^−1^ silicate rate, while the lowest values was 0.815 kg m^−3^ under the four time irrigation treatment with zero-silicate application.

## 3. Discussion

The ideal ratios of C:N:P were previously presented as 106:16:1 as evaluated in the aquatic ecosystems [24]. Soils with N/P ratios below 14 can be classified as nitrogen-limited, presenting very low contents of soil N forms [25].

Soil salinity and soil alkalinity were negatively correlated (r = −0.75, *p* < 0.01) across the irrigation treatments. Significant variation in soil nutrient dynamics was observed among the irrigation treatments, suggesting that the notable increase in soil water retention enhanced soil buffering capacity. This, in turn, led to heightened soil microbial activity and improved the cycling of soil nutrients in the upper soil horizons [26].

In the context of the factor analysis results, it becomes evident that changes in soil salinity and cation exchange capacity may exert a notable influence on soil respiration capacity and carbon loss. The observed negative loadings for soil respiration (CO_2_), carbon loss as C-CO_2_, and C-CO_2_/SOC ratio in relation to factors associated with soil salinity and cation exchange capacity support this notion.

These findings suggest that increased soil salinity and cation exchange capacity may negatively impact soil respiration, leading to elevated carbon loss from soil. This could be attributed to several factors, including reduced microbial activity and organic matter decomposition in saline soils, which may inhibit the release of CO_2_ through respiration processes. Additionally, the higher cation exchange capacity in these soils may result in increased adsorption of organic carbon, reducing its availability for microbial decomposition and subsequent CO_2_ release.

The implications of these relationships are significant for soil management and carbon cycling in agricultural ecosystems. High soil salinity and cation exchange capacity could potentially compromise soil health and fertility by reducing microbial activity and organic matter decomposition rates. As a result, strategies aimed at mitigating soil salinity and optimizing cation exchange capacity may be crucial to maintaining soil respiration capacity and minimizing carbon loss [27].

The dataset shows a substantial correlation among the variables of soil pH, soil organic carbon (SOC), total nitrogen, molar C/P and N/P ratios, and soluble and effective sodium, as indicated by their high positive loadings (>0.50). This correlation could suggest common underlying mechanisms or environmental factors. For example, the presence of greater soil pH, SOC, and total nitrogen loadings may indicate that regions with elevated pH levels also exhibit increased levels of organic carbon and nitrogen. This correlation could be suggestive of more fertile or healthier soils. 

In addition, the molar C/P and N/P ratios offer valuable information about nutrient cycling and stoichiometry in the soil system. Significant positive loadings for these ratios suggest a possible equilibrium or imbalance in the availability of carbon, phosphorus, and nitrogen, which could impact the dynamics of nutrients and the productivity of the ecosystem. 

In contrast, variables such as the dissolved proportion of SOC represented as DOC/SOC, total phosphorus, bicarbonates, chlorine, SO4, and effective calcium showed significant negative loadings (>−0.50). The negative loadings indicate a negative correlation between these variables and the components with positive loadings. For instance, the presence of negative loading for total phosphorus and bicarbonates could suggest reduced quantities of these constituents in regions characterized by elevated soil pH and organic carbon content [28].

Furthermore, the adverse impact of effective calcium, bicarbonates, chlorine, and SO_4_ loadings suggests the possibility of interactions or competition among these ions in the soil system. Gaining a comprehensive understanding of these connections is essential to deciphering the intricate dynamics of soil chemistry and its consequences for the functioning of ecosystems. 

These findings indicate that irrigation can affect the processes involved in the movement of carbon, nitrogen, and phosphorus in soil by altering the levels of anions and alkalinity. These findings emphasize the significance of taking into account not only individual soil qualities but also their interconnections and potential drivers, such as irrigation techniques, in attempts to manage soil and conserve the environment. Additional investigation and analysis of these connections could offer significant knowledge for enhancing agricultural methods and reducing environmental consequences [29,30].

The third factor explained 9% of the total variance with high negative loadings (>−0.50) from soil moisture, soil organic carbon, dissolved organic carbon, total nitrogen, carbon loss, and C-CO_2_/SOC, directly indicating that the number of irrigation applications, when affecting soil moisture, directly influence the dynamic changes in soil carbon preservation and mineralization processes.

The highest communality values were for soil moisture, soil organic reserve parameters, soil carbon loss assessment parameters, and soluble cations, pointing out that these parameters were the most affected by the changes in irrigation rates. In dry areas, irrigation rates, which influence soil moisture regimes, tend to develop soil organic dynamics parameters and thus reduce soil carbon loss [31].

The number of irrigation applications plays a crucial role in the dynamics of soil organic matter for its storage and mineralization processes because of soil response to wetting/drying cycles. The highest contribution to the first two factors was from the four times irrigation treatment at each sampling time (Figure 2b). The increasing factor loadings of CEC and soil cations observed for the first factor, as well as soil organic C and N for the second factor, suggest the dynamic stability of soil organic matter contents (Figure 2a).

This enhancement in wheat crop yield due to increasing irrigation frequency aligns with the findings of the study in [32].

Grain yield, biological yield, and straw yield showed the highest significant increase (*p* < 0.05) under the three times irrigation (I3) treatment because of the soil’s physical and chemical development at this irrigation rate. These results agree with those of [33], corroborating that silicon helps the plant to alleviate drought stress, thus enhancing crop productivity. Likewise, [34] revealed that foliar silicon application improved grain yield in drought-stressed wheat crops by further decreasing canopy temperature. It was also applied in priming seeds, which demonstrated an improvement in yield parameters in wheat crops, whose seeds were treated with silicon during seed preparation, which triggered water-deficit tolerance until plant maturity.

The effect size statistics revealed that the irrigation factor was more affected than the sodium silicate factor in all the studied parameters, even when the two factors were significant. This was obvious in grain yield and harvest index, where both irrigation and sodium silicate and their interaction were significantly affected. The most responsive parameter was grain yield, where the eta squared ($\eta^{2}$) values were 0.701 and 0.082, the partial eta squared ($\eta_{p}^{2}$) values were 0.946 and 0.672, the omega squared ($\omega^{2}$) values were 0.692 and 0.077, the partial omega squared ($\omega_{p}^{2}$) values were 0.885 and 0.461, and the epsilon squared ($\varepsilon^{2}$) values were 0.694 and 0.077 for irrigation and sodium silicate, respectively.

Additionally, the weight of 1000 grains (grain index) (0.38) showed significant influence on grain yield. These results are in agreement with the results of [35], who reported that a 20% reduction in irrigation requirements did not significantly affect plant height, spike length, the number of spikelets/spike, biological yield (tons/fed.), straw yield (tons/fed.), or harvest index. However, the number of grains per spike, grain weight per spike, and grain yield (tons per feddan) were significantly impacted.

Also, [36] reported that foliar potassium silicate rates improved drought tolerance in different growth stages by enhancing the morphological, physiological, and antioxidant potential of the wheat crop.

Silicon may be a low-cost way to reduce crop water stress. However, its effectiveness varies greatly, and the basic processes are unknown. Of concern is whether and how variability in plant silicon accumulation is related to its effects on plant growth during water stress.

Previous research has examined variations in silicon accumulation and their effects on plant growth and crop yield [37,38]. Regarding the relationship between different accumulation of silicon and the variations in stress tolerance, researchers investigated whether silicon affects osmotic and drought stresses differently in strains with higher silicon content compared with those with lower content.

Some investigations indicated that silicon enhances wheat growth under osmotic and drought stress [2,10], but other studies did not find a significant increase in shoot dry weight biomass [20,39]. Furthermore, in many other crops, such as soybean, growth has been reported to show no response to silicon under drought despite the reduced membrane damage and increased peroxidase activity. In barley, silicon reduced osmotic stress but did not affect biomass [18,19]. These discrepancies could be partially clarified by methodological differences; for example, many research studies used sodium and potassium silicate as a silicon treatment but did not account for cation concentrations in the control treatment. As a result, the demonstrated silicon response was due to additional sodium and potassium. Another possibility of misunderstanding is the difference in interpretation; many researchers have reported that silicon positively affects tolerance to osmotic or drought stress, which can be explained when compared with control treatments with a higher response to the associated stress. Another significant component may be the genotype’s silicon response. The authors of [40] realized that silicon positively affected growth in a poinsettia cultivar. Additionally, sugarcane response to silicon varied significantly in dry weight only in one of four cultivars under drought conditions [17]. The present study states that foliar application of silicon may enhance plant resistance to water deficit. However, more detailed studies with more levels of both irrigation and silicon application should be conducted to achieve comprehensive conclusions about the role of silicon.

## 4. Materials and Methods

### 4.1. Study Area

**Figure 5:**
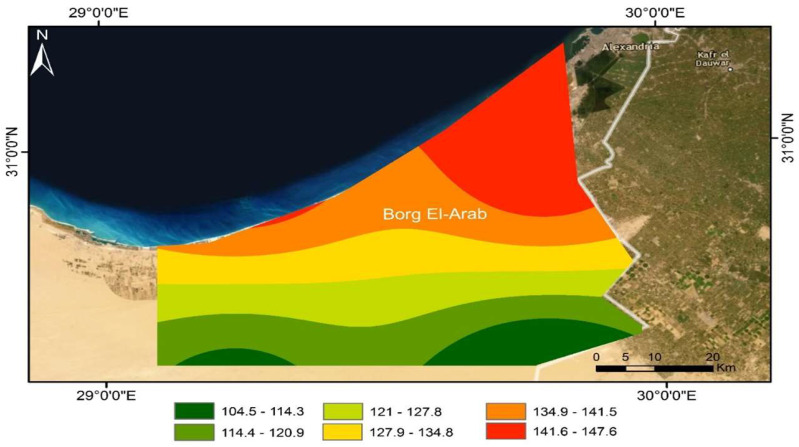
Annual rainfall (mm) in the study area.

The research site was situated in New Borg El-Arab City, within Alexandria Governorate, Egypt, approximately 10 km inland from the sea (see Figure 5). It is located at latitude 30.91 N, longitude 29.68 E, at an altitude of 54 m above sea level. Its location is representative of the arid climate of the northwestern coast of Egypt.

**Figure 6:**
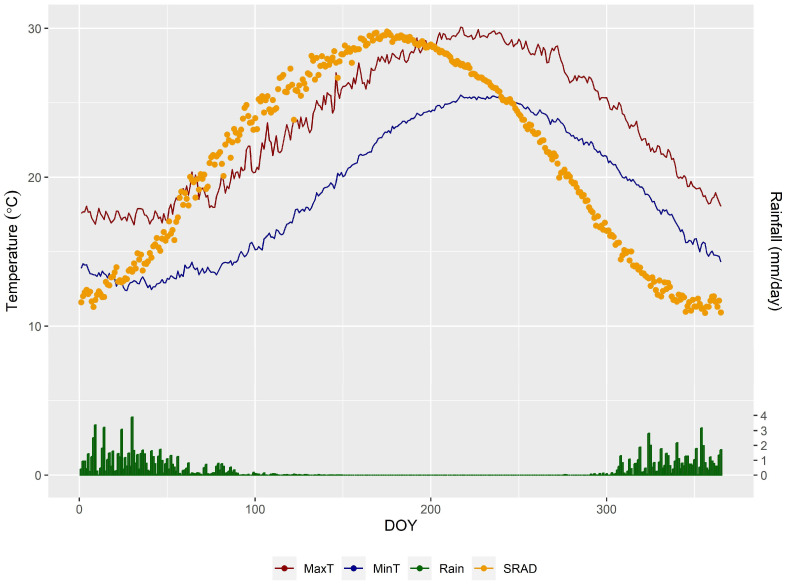
Weather data (rain in mm, max and min temperature in °C, and solar radiation in MJm^−2^). Source: https://power.larc.nasa.gov (accessed on 15 March 2024).

The arid climate of the area is typified by scorching, dry summers and mild winters, with annual precipitation ranging from 100 to 150 mm (see Figure 6). Short rainstorms sometimes occur, mainly in winter, most of which fall in January, February, and March. The Egyptian coastal zones receive noticeable amounts of rainfall, especially in winter; therefore, precipitation is considered the main source of groundwater recharge for aquifers in the area. Figure 5 represents the mean annual rainfall map during 2011–2020 according to the CRU TS (Climatic Research Unit gridded Time Series) dataset [41] and the World Imagery with Metadata web map (2023) as basemap Imagery with Metadata—Overview (https://www.arcgis.com/). The field experiment spanned two wheat growing seasons, taking place from November to May in both the years 2021/2022 and 2022/2023. During the two seasons, the highest temperature was 37 ± 5 °C in May, while the lowest was 10 ± 4 °C in January. The average humidity varied between 60 and 70%. The average wind speed was around 14 km h^−1^.

### 4.2. Field Experiment

The field experiment was designed as a split-plot design, comprising three replications. Irrigation treatments were assigned to the main plots, whereas foliar silicon spraying was assigned to the sub-plots as subsequently explained. The irrigation treatments were applied as follows: (i) one time irrigation (I1) (1000 m^3^ ha^−1^) at sowing in mid-November, (ii) two times irrigation (I2) (2000 m^3^ ha^−1^) at planting and tillering, (iii) three times irrigation (I3) (3000 m^3^ ha^−1^) at planting, tillering, and elongation, (iv) four times irrigation (I4) (4000 m^3^ ha^−1^) at planting, tillering, elongation, and grain filling. Irrigation treatments were sequenced by accumulating the number of irrigation applications per 1000 m^3^ ha^−1^. Three different sodium silicate (Na_2_SiO_3_) (purchased from Al-Gomhoria company, Alexandria, Egypt) concentrations (0, 200, and 400 mg L^−1^) were applied one month after planting and repeated after two weeks. Each sub-plot area was delimited at 6 m^2^ (2 × 3 m) and divided into six rows, separated by a 30 cm distance. Wheat grains cv. Giza 168 (pedigree: MIL/BUC//Seri CM93046-8M-0Y-0M-2Y-0B-0SH) were sourced from the Agriculture Research Center, Giza, Egypt, and were manually drilled into the rows in mid-November for both seasons at the rate of 143 kg ha^−1^. The planting was carried out at 1 cm soil depth in mid-November, and harvest was in late April for each season. The experimental fields were fertilized with 357 kg ha^−1^ phosphorus (15.5% P_2_O_5_), during seedbed preparation, mixed thoroughly into the soil before sowing, and then gently raked to a depth of 10–15 cm. Nitrogen fertilizer (ammonium nitrate of 33.5% N) was administered at a rate of 238 kg/ha in three equal doses at planting, tillering, elongation, and during rainfall.

### 4.3. Soil Quality Assessment

Soil samples were collected in triplicate from each irrigation plot, with sampling being conducted three times at two-month intervals. Fresh soil cores were collected from each plot, in triplicates, to determine soil moisture contents by using the gravimetric method. The collected samples were oven-dried at 105 °C for 24 h. Initial soil characteristics were analyzed before the experiment (Table 9). The soil samples were air-dried and then ground and sieved through a 2 mm mesh filter for subsequent soil analyses [42]. By using Robinson’s pipette method, soil particle analysis was performed (Eijkelkamp Agri research Equipment, Giesbeek, The Netherlands). Based on the soil particle size analysis, the soil texture was classified as sandy clay loam (62.43%), silt (13.02%), and clay (24.55%). The total CaCO_3_ content was quantified by CO_2_ release from fine soil upon acidification with 10% HCl. Soil pH and electrical conductivity (EC) were measured in 1:2.5 *w*/*v* and 1:1 *w*/*v* soil aqueous extracts, respectively. Soluble cations (Na^+^, K^+^, Ca^2+^, and Mg^2+^) and anions (SO_4_^2−^, Cl^−^, and HCO_3_^−^) were determined in the 1:1 soil aqueous extract. Soluble Na^+^ and K^+^ were estimated (flame photometer, PG Instruments M: FP902). The EDTA titration method measured soluble Ca^2+^ and Mg^2+^ in the extract. Soluble SO_4_^2−^ was determined by barium sulfate precipitation. Soluble Cl^−^ and HCO_3_^−^ were determined by titration with silver nitrate and 0.01 N H_2_SO_4_, respectively. Soil organic carbon (SOC) was assessed via the Walkley–Black wet oxidation method. Dissolved organic carbon (DOC) was extracted from a 1:5 (*w*/*v*) suspension of deionized water at 25 °C. Following a 24 h shaking period, the suspension underwent filtration through a cellulose nitrate membrane filter with a pore size of 0.45 μm. DOC, representing the dissolved fraction of soil organic matter (SOM) in the resulting aqueous extract, was quantified by using a total organic carbon (TOC) analyzer (Torch Combustion TOC/TN Analyzer; Teledyne Tekmar, OHIO, USA) [43]. The dissolved organic carbon fraction was expressed as a percent (DOC/SOC; %) of total SOC content. Total nitrogen (TN) was measured by using the Kjeldahl method. Available phosphorus was extracted with 0.5 M NaHCO_3_ at pH 8.5 according to [44] and measured by the PG Instruments T80 UV/VIS Spectrophotometer. The stoichiometric balance of soil C:N:P ratio, known as the “Redfield ratio”, was also calculated considering the molar ratios of C, N, and P [25]. The cation exchange capacity (CEC) was also assessed, including the effective percentage of each cation concentration in cmol kg^−1^, presented as Na _effective_, K _effective_, Ca _effective_, and Mg _effective_.

The soda lime absorption method was applied for soil respiration measurements (the metabolically emitted CO_2_ from the soil) by using the dark cover box technique in the field. The detected CO_2_ concentrations were calculated after 24 h of incubation of soda lime on the soil surface in the delimited area under the cover boxes installed on the soil surface.

The calculations were derived after the oven-drying of soda lime, followed by multiplication by a correction factor of 1.69, and presented in mg g^−1^ soil. The soil carbon loss emitted into the atmosphere as C-CO_2_ was calculated by multiplying the CO_2_ readings by 12/44 (the molecular weight of C/CO_2_) and then expressed in mg g^−1^.

### 4.4. Wheat Crop Yield Assessment

Plants were harvested and randomly chosen from each sub-plot to assess wheat yield measurements by measuring the LAI by using the disc method [45] the number of grains/spike and grain weight/spike (g) by taking the average of 10 random spikes from each plot; the number of spikes m^−2^ by counting the spikes in a random square meter in each plot; grain yield (ton ha^−1^) by weighing the grains threshed from the plot and calculating the yield for hectare; straw yield (ton ha^−1^) as the difference between biological yield and grain yield; biological yield (ton ha^−1^) by weighing all the plants from the harvested plot; 1000 grain wt. (gm); and the harvest index (HI) by dividing the grain yield by the biological yield.

Ref. [46] linked the relative yield decrease with the relative evapotranspiration deficit by using the following equation:

(1) $$1-\frac{Y_{a\;}}{Y_{m}}=K_{y}(1-\frac{{ET}_{a\;}}{{ET}_{m}})$$

where ET_a_ represents the rate of actual evapotranspiration, quantifying the water stress in plants (actual water consumption; mm), and ET_m_ denotes the rate of maximum evapotranspiration (maximum water consumption; mm). The relative yield, Yr, is calculated as Y_a_/Y_m_, where Y_a_ is the actual yield and Y_m_ is the maximum yield. When there is any reduction in irrigation, Y_a_ equals Y_m_. K_y_ represents the yield response factor.

### 4.5. Water Productivity Assessment

#### 4.5.1. Applied Irrigation Water (AIW)

The recommended amount of applied irrigation water was calculated as described by [47] as follows:

(2) $$AIW\;(mm\;{day}^{-1})=\frac{ETc}{Ea}$$

where Ea represents the irrigation efficiency (0.60 for surface irrigation).

#### 4.5.2. Crop Evapotranspiration (ETc)

The FAO crop evapotranspiration method was used to calculate crop water requirements (Table 2) by using reference evapotranspiration (ETo; mm) and crop coefficient (Kc) as described by [48]:

(3) $$ETc\;(mm\;{day}^{-1})=ETo\times Kc$$

The ETo was calculated by using CROPWAT software based on the FAO Penman–Monteith equation [49].

#### 4.5.3. Irrigation Water Productivity (IWP)

IWP (irrigation water productivity; kg m^−3^) calculations followed [50] method as follows:

(4) $$IWP=\frac{Y_{a}}{AIW}$$

where Y_a_ is the seed yield of various treatments (kg ha^−1^) and AIW is the seasonal amount of applied water (m^3^ ha^−1^), which was calculated by using the following equation:

(5) AW=AIW+Peff+S

where AIW denotes the applied irrigation water, Peff denotes adequate rainfall during the growing season, and S denotes groundwater contribution to crop water use (neglected because the water table was deeper than 2.0 m) [51].

### 4.6. Statistical Analysis

Following the assessment of error homogeneity through Bartlett’s test, a combined analysis encompassing both seasons was undertaken. Soil quality parameters together with wheat yield assessment parameters were compared among all applied treatments by using Tukey’s HSD (Honestly Significant Difference) tests at the *p* < 0.05 level, following ANOVA performed by using STATISTICA 10 by StatSoft, Inc. (Tulsa, OK, USA) [52]. The statistical analysis of wheat and grain yield assessment parameters was conducted by using R Statistical Software (R_Core_Team, 2021).

## 5. Conclusions

The results confirm that three times irrigation application amounting to 3000 m^3^ ha^−1^ for wheat in these soils as proposed can lead to the highest nutrient retention, wheat yield, and water productivity. This irrigation rate showed adequate soil carbon storage capacity against carbon loss that was highly dependent on the solubility of soil cations and anions, thus affecting soil microbial activity. The results also show that the changes in soil organic matter dynamics in preservation and mineralization across the different irrigation times in these calcareous soils varied due to soil salinity and alkalinity and soil cation exchange capacity. Further research is needed to identify the soil organic matter fractions more susceptible to the changes in soil moisture regimes in these calcareous soils with poor structure.

The three times irrigation rate represents 60% of the recommended total applied irrigation water (5000 m^3^ ha^−1^) during the wheat season and produced a high grain yield and high water productivity on the northwest coast of Egypt. Moreover, foliar application of silicon can alleviate the adverse effect of water deficit in wheat production. Finally, a high yield of wheat grains can be achieved under deficit irrigation on the northwest coast of Egypt with a dry climate by spraying 400 mg L^−1^ sodium silicate. Deficit irrigation can be substituted by foliar silicon application, which can be, in addition, considered for climate-smart plants’ long-term productivity under drought stress conditions. Further investigation is required to comprehend the dynamic mechanisms of silicate in different crops under diverse climatic conditions, which influence irrigation regimes.

## Figures and Tables

**Table 1:** General soil characteristics, as well as CO_2_ emissions from soil and soil carbon loss.

Treatments		SM (%)	pH	EC (dS m^−1^)	SOC (%)	DOC (mg L^−1^)	DOC/SOC (%)
1st month	I1	20.36 ± 0.34 f	8.19 ± 0.20 a	2.35 ± 0.11 ab	1.32 ± 0.12 ab	17.00 ± 1.32 a	0.64 ± 0.00 c
	I2	22.78 ± 0.65 de	8.17 ± 0.12 a	2.20 ± 0.12 b	1.22 ± 0.03 ab	20.08 ± 1.24 a	0.83 ± 0.01 a
	I3	22.72 ± 0.55 de	8.12 ± 0.24 a	2.95 ± 0.13 a	1.14 ± 0.08 b	18.64 ± 1.32 a	0.82 ± 0.01 a
	I4	23.37 ± 0.34 d	8.21 ± 0.23 a	2.16 ± 0.20 b	1.58 ± 0.16 ab	20.27 ± 2.22 a	0.64 ± 0.00 c
3rd month	I1	21.27 ± 0.45 ef	8.54 ± 0.21 a	0.62 ± 0.23 d	1.39 ± 0.03 ab	19.82 ± 1.84 a	0.71 ± 0.01 b
	I2	23.53 ± 0.91 d	8.70 ± 0.22 a	0.46 ± 0.21 d	1.36 ± 0.24 ab	19.73 ± 1.02 a	0.72 ± 0.01 b
	I3	25.54 ± 0.75 bc	8.50 ± 0.18 a	0.86 ± 0.21 cd	1.33 ± 0.32 ab	21.50 ± 1.52 a	0.81 ± 0.01 a
	I4	27.00 ± 0.53 ab	8.57 ± 0.16 a	0.59 ± 0.23 d	1.72 ± 0.22 ab	20.17 ± 1.63 a	0.59 ± 0.01 d
5th month	I1	20.73 ± 0.45 f	8.37 ± 0.17 a	0.94 ± 0.32 cd	1.51 ± 0.34 ab	22.11 ± 1.24 a	0.73 ± 0.01 b
	I2	25.31 ± 0.19 c	8.49 ± 0.24 a	0.80 ± 0.30 cd	1.34 ± 0.54 ab	21.80 ± 1.67 a	0.81 ± 0.01 a
	I3	26.63 ± 0.28 abc	8.52 ± 0.17 a	1.02 ± 0.24 cd	1.42 ± 0.23 ab	22.87 ± 1.34 a	0.81 ± 0.01 a
	I4	28.12 ± 0.65 a	8.29 ± 0.16 a	1.44 ± 0.25 c	1.88 ± 0.06 a	22.36 ± 1.63 a	0.60 ± 0.01 d
**Treatments**		**TN** **(%)**	**TP** **(%)**	**Molar** **C/P Ratio**	**Molar** **N/P Ratio**	**CO_2_ Emissions** **(mg g^−1^)**	**C-CO_2_** **Loss** **(mg g^−1^)**
11st month	I1	0.06 ± 0.01 e	0.12 ± 0.01 a	29.11 ± 2.22 ef	1.13 ± 0.34 ef	0.11 ± 0.01 d	0.03 ± 0.00 e
	I2	0.06 ± 0.02 e	0.12 ± 0.01 a	25.29 ± 2.23 f	1.05 ± 0.31 f	0.11 ± 0.01 d	0.03 ± 0.00 e
	I3	0.07 ± 0.01 de	0.12 ± 0.02 a	24.96 ± 2.32 f	1.22 ± 0.24 ef	0.13 ± 0.01 cd	0.04 ± 0.00 d
	I4	0.09 ± 0.02 de	0.10 ± 0.01 a	39.98 ± 2.21 cd	1.86 ± 0.31 def	0.10 ± 0.01 d	0.03 ± 0.00 e
3rd month	I1	0.11 ± 0.02 cde	0.11 ± 0.01 a	33.18 ± 2.65 de	2.16 ± 0.22 cd	0.16 ± 0.01 bc	0.04 ± 0.00 d
	I2	0.11 ± 0.02 cde	0.10 ± 0.01 a	36.56 ± 2.27 d	2.48 ± 0.21 cd	0.17 ± 0.01 b	0.05 ± 0.00 c
	I3	0.11 ± 0.03 cde	0.12 ± 0.01 a	28.81 ± 2.26 ef	2.02 ± 0.25 cde	0.15 ± 0.01 bc	0.04 ± 0.00 d
	I4	0.18 ± 0.02 ab	0.10 ± 0.01 a	44.25 ± 2.73 bc	3.88 ± 0.35 b	0.18 ± 0.01 b	0.05 ± 0.00 c
5th month	I1	0.12 ± 0.01 bcde	0.09 ± 0.01 a	44.58 ± 2.56 bc	2.99 ± 0.42 c	0.24 ± 0.02 a	0.06 ± 0.00 b
	I2	0.13 ± 0.02 bcd	0.07 ± 0.01 a	49.84 ± 2.54 b	4.18 ± 0.45 b	0.23 ± 0.01 a	0.06 ± 0.00 b
	I3	0.16 ± 0.03 abc	0.08 ± 0.01 a	48.02 ± 2.65 b	4.59 ± 0.31 b	0.26 ± 0.01 a	0.07 ± 0.00 a
	I4	0.20 ± 0.03 a	0.07 ± 0.01 a	67.37 ± 2.74 a	6.07 ± 0.09 a	0.25 ± 0.02 a	0.07 ± 0.00 a

EC: electrical conductivity; SOC: soil organic carbon; DOC: dissolved organic carbon; I1: one time irrigation; I2: two times irrigation; I3: three times irrigation; I4: four times irrigation. Different letters indicate significant data variability; means with the same letter are not significantly different from each other at α < 0.05 as checked by Tukey’s HSD test among all irrigation treatments. The same letters indicate no significance. TN: total nitrogen; TP: total phosphorus; CO_2_: emitted CO_2_ from the soil as soil respiration; C-CO_2_: soil carbon loss as C-CO_2_.

**Table 2:** Soluble cations and anions in soil under irrigation treatments.

Treatments		Soluble Cations (mg kg^−1^)				Soluble Anions (mg kg^−1^)		
		Na^+^	K^+^	Ca^2+^	Mg^2+^	HCO_3_^−^	Cl^−^	SO_4_^2−^
1st month	I1	5.29 ± 1.65 c	44.01 ± 2.45 b	23.27 ± 1.39 ab	9.14 ± 1.64 abc	467.13 ± 5.64 a	207.68 ± 2.35 a	454.69 ± 2.67 a
	I2	5.29 ± 1.12 c	43.86 ± 3.21 b	23.27 ± 1.64 ab	9.11 ± 1.64 abc	563.85 ± 6.34 a	218.24 ± 2.19 a	483.84 ± 3.07 a
	I3	7.21 ± 1.35 bc	53.46 ± 3.35 a	26.58 ± 1.63 a	11.10 ± 1.27 ab	563.65 ± 5.36 a	218.29 ± 2.88 a	483.82 ± 3.64 a
	I4	9.12 ± 1.23 abc	58.58 ± 2.24 a	27.07 ± 1.34 a	12.17 ± 1.36 a	406.21 ± 3.09 a	168.01 ± 2.87 a	281.58 ± 2.67 a
3rd month	I1	8.09 ± 1.22 bc	35.83 ± 2.27 cd	20.73 ± 1.32 bc	7.44 ± 1.63 bcd	434.39 ± 3.34 a	172.95 ± 2.69 a	292.57 ± 2.64 a
	I2	8.86 ± 1.64 abc	41.01 ± 2.26 bcd	21.17 ± 1.36 bc	8.52 ± 1.53 abcd	434.04 ± 3.64 a	172.84 ± 2.91 a	292.48 ± 3.04 a
	I3	8.88 ± 1.52 abc	41.40 ± 2.35 bcd	21.17 ± 1.53 bc	8.60 ± 1.34 abcd	444.83 ± 5.65 a	186.40 ± 2.73 a	332.94 ± 3.31 a
	I4	10.67 ± 1.33 ab	43.14 ± 3.01 bc	22.31 ± 1.55 bc	8.96 ± 1.35 abcd	378.31 ± 5.01 a	146.25 ± 2.74 a	243.12 ± 3.36 a
5th month	I1	12.94 ± 1.68 a	23.74 ± 2.21 e	18.35 ± 1.32 c	4.93 ± 1.08d	378.32 ± 4.34 a	146.27 ± 2.64 a	242.98 ± 2.06 a
	I2	12.97 ± 1.69 a	24.07 ± 2.03 e	18.33 ± 1.24 c	5.00 ± 1.04 cd	391.60 ± 3.68 a	162.40 ± 2.64 a	260.21 ± 2.27 a
	I3	10.88 ± 1.56 ab	35.20 ± 2.05 d	19.37 ± 1.32 bc	7.31 ± 1.54 bcd	400.58 ± 3.37 a	172.15 ± 2.34 a	274.74 ± 3.15 a
	I4	11.21 ± 1.67 ab	38.13 ± 2.01 bcd	20.50 ± 1.09 bc	7.92 ± 1.35 bcd	400.76 ± 2.99 a	171.96 ± 2.77 a	274.31 ± 2.68 a

I1: one time irrigation; I2: two times irrigation; I3: three times irrigation; I4: four times irrigation. Different letters indicate significant data variability; means with the same letter are not significantly different from each other at α < 0.05 as checked by Tukey’s HSD test among all irrigation treatments. The same letters indicate no significance.

**Table 3:** Cation exchange capacity (CEC) and effective percentages of exchangeable cations (Na^+^, K^+^, Ca^2+^, and Mg^2+^) for the studied soil treatments.

Treatments		CEC (cmol kg^−1^)	Na^+^_Effective_	K^+^_Effective_	Ca^2+^_Effective_	Mg^2+^_Effective_
			%			
1st month	I1	3.50 ± 0.02 ab	13.13±2.14 a	32.20±2.34 a	33.20 ± 1.07 a	21.47 ± 1.67 a
	I2	3.50 ± 0.31 ab	13.15 ± 1.98 a	32.15 ± 2.64 a	33.27 ± 2.15 a	21.43 ± 1.64 a
	I3	4.24 ± 0.31 ab	14.78 ± 1.64 a	32.33 ± 2.35 a	31.34 ± 1.87 a	21.55 ± 1.34 a
	I4	4.65 ± 0.36 a	17.05 ± 1.35 a	32.30 ± 3.08 a	29.11 ± 1.67 a	21.54 ± 1.08 a
3rd month	I1	3.27 ± 0.34 ab	21.51 ± 2.22 a	28.09 ± 3.04 a	31.68 ± 2.01 a	18.73 ± 1.67 a
	I2	3.58 ± 0.38 ab	21.51 ± 2.04 a	29.36 ± 2.01 a	29.55 ± 2.00 a	19.58 ± 1.44 a
	I3	3.60 ± 0.97 ab	21.46 ± 2.00 a	29.49 ± 1.06 a	29.40 ± 1.79 a	19.66 ± 1.37 a
	I4	3.89 ± 0.62 ab	23.88 ± 1.98 a	28.46 ± 1.87 a	28.69 ± 1.67 a	18.97 ± 1.67 a
5th month	I1	3.06 ± 0.31 b	36.81 ± 1.79 a	19.90 ± 2.06 a	30.01 ± 2.01 a	13.27 ± 1.35 a
	I2	3.07 ± 0.64 b	36.70 ± 2.03 a	20.08 ± 2.34 a	29.83 ± 2.07 a	13.39 ± 1.33 a
	I3	3.42 ± 0.64 ab	27.67 ± 2.65 a	26.40 ± 2.09 a	28.33 ± 1.87 a	17.60 ± 1.97 a
	I4	3.63 ± 0.36 ab	26.86 ± 2.08 a	26.94 ± 2.13 a	28.24 ± 1.67 a	17.96 ± 1.64 a

CEC: cation exchange capacity; I1: one time irrigation; I2: two times irrigation; I3: three times irrigation; I4: four times irrigation. Different letters indicate significant data variability; means with the same letter are not significantly different from each other at α < 0.05 as checked by Tukey’s HSD test among all irrigation treatments. The same letters indicate no significance.

**Table 4:** First three-factor structures with communality values of factor analysis.

Soil Variables	Factor 1	Factor 2	Factor 3	Communality
SM	−0.091	0.148	−0.938	0.91
pH	−0.404	0.518	0.266	0.50
EC	0.608	−0.431	0.270	0.63
SOC	0.111	0.719	−0.547	0.83
DOC	−0.466	0.229	−0.789	0.89
DOC/SOC	−0.483	−0.643	−0.137	0.67
TN	−0.222	0.764	−0.510	0.89
TP	0.340	−0.757	−0.086	0.70
C/P	−0.178	0.828	−0.291	0.80
N/P	−0.279	0.760	−0.410	0.82
CO_2_	−0.616	0.330	−0.691	0.97
C−CO_2_	−0.616	0.330	−0.691	0.97
C−CO_2_/SOC	−0.726	0.080	−0.623	0.92
Na^+^	−0.524	0.741	−0.210	0.87
K^+^	0.951	−0.172	0.166	0.96
Ca^2+^	0.891	−0.281	0.216	0.92
Mg^2+^	0.951	−0.172	0.166	0.96
HCO_3_^−^	0.332	−0.863	0.115	0.87
Cl^−^	0.388	−0.795	0.189	0.82
SO_4_^2−^	0.376	−0.831	0.217	0.88
CEC	0.913	0.097	0.123	0.86
Na^+^_Effective_	−0.772	0.545	−0.195	0.93
K^+^_Effective_	0.857	−0.400	0.159	0.92
Ca^2+^_Effective_	−0.012	−0.848	0.237	0.78
Mg^2+^_Effective_	0.857	−0.400	0.159	0.92
Variation (%)	59	17	9	
Total variance (%)	59	76	85	85

SM: soil moisture; EC: electrical conductivity; SOC: soil organic carbon; DOC: dissolved organic carbon; TN: total nitrogen; TP: total phosphorus; CEC: cation exchange capacity.

**Table 5:** Wheat yield assessment under irrigation and sodium silicate treatments and their interaction with the five effect size statistics tested by using R-2021software.

	Treatments	Grain No./Spike	Grain wt./Spike (g)	Spikes (m^2^)	LAI	1000 Grain Weight (g)	Grain Yield (ton/ha)	Biological Yield (ton/ha)	Straw Yield (ton/ha)	HI
Irrigation (I)	I1	32.930 b	1.040 b	402.220 b	1.580 b	3.160 a	2.100 b	10.290 c	8.170 b	0.200 b
	I2	35.980 ab	1.050 b	472.890 ab	2.460 a	3.080 a	2.500 b	12.790 b	10.290 a	0.200 b
	I3	42.840 ab	1.350 a	454.220 ab	2.390 a	3.220 a	4.430 a	15.190 a	10.790 a	0.290 a
	I4	44.970 a	1.530 a	521.330 a	2.190 ab	3.670 a	4.360 a	14.950 a	10.600 a	0.290 a
Significance of irrigation		*	**	*	*	NS	***	***	**	**
Effect size statistics for irrigation	$\eta^{2}$	0.530	0.590	0.308	0.494	0.132	0.701	0.658	0.452	0.474
	$\eta_{p}^{2}$	0.770	0.709	0.582	0.694	0.259	0.946	0.836	0.622	0.842
	$\omega^{2}$	0.496	0.537	0.263	0.447	0.060	0.692	0.629	0.394	0.455
	$\omega_{p}^{2}$	0.584	0.500	0.349	0.481	0.067	0.885	0.686	0.394	0.696
	$\varepsilon^{2}$	0.500	0.545	0.267	0.453	0.061	0.694	0.634	0.401	0.457
Silicon (Si)	0 mg L^−1^	38.420 a	1.260 a	476.670 a	2.120 a	3.280 a	2.880 b	12.570 a	9.690 a	0.220 b
	200 mg L^−1^	37.970 a	1.230 a	474.000 a	2.080 a	3.420 a	3.380 a	13.760 a	10.380 a	0.240 b
	400 mg L^−1^	39.830 a	1.250 a	437.330 a	2.250 a	3.150 a	3.760 a	13.570 a	9.810 a	0.270 a
Significance of silicon		NS	NS	NS	NS	NS	***	NS	NS	***
Effect size statistics for silicon	$\eta^{2}$	0.008	0.001	0.008	0.021	0.030	0.082	0.046	0.037	0.113
	$\eta_{p}^{2}$	0.045	0.005	0.035	0.090	0.073	0.672	0.261	0.118	0.561
	$\omega^{2}$	0.000	0.000	0.000	0.000	0.000	0.077	0.029	0.002	0.102
	$\omega_{p}^{2}$	0.000	0.000	0.000	0.000	0.000	0.461	0.092	0.004	0.339
	$\varepsilon^{2}$	0.000	0.000	0.000	0.000	0.000	0.077	0.029	0.002	0.102
Significance of interaction		NS	NS	**	NS	NS	**	NS	NS	*
Effect size statistics for interaction	$\eta^{2}$	0.115	0.054	0.320	0.090	0.088	0.150	0.101	0.122	0.272
	$\eta_{p}^{2}$	0.421	0.183	0.591	0.294	0.189	0.790	0.438	0.307	0.754
	$\omega^{2}$	0.055	0.000	0.234	0.009	0.000	0.135	0.052	0.019	0.237
	$\omega_{p}^{2}$	0.135	0.000	0.323	0.018	0.000	0.601	0.153	0.030	0.545
	$\varepsilon^{2}$	0.056	0.000	0.237	0.009	0.000	0.135	0.052	0.019	0.239

LAI: leaf area index; HI: harvest index; I1: one time irrigation; I2: two times irrigation; I3: three times irrigation; I4: four times irrigation. ***, *p*-value < 0.001; **, *p*-value < 0.01; *, *p*-value < 0.05; and NS, *p*-value > 0.05. Means followed by same letters are not significantly different.

**Table 6:** Grain yield, harvest index, and number of spikes/m^2^ of wheat under irrigation and silicate treatments.

Irrigation	Silicon	Grain Yield (ton ha^−1^)	Harvest Index	Number of Spikes (m^2^)
I1	0 mg L^−1^	0.95 d	0.11 e	378.67 b
	200 mg L^−1^	2.40 c	0.21 bcd	392.0 b
	400 mg L^−1^	2.98 c	0.28 abc	404.0 ab
I2	0 mg L^−1^	2.86 c	0.22 bcd	520.0 ab
	200 mg L^−1^	2.17 c	0.17 de	413.3 ab
	400 mg L^−1^	2.48 c	0.20 cd	564.0 ab
I3	0 mg L^−1^	4.48 a	0.30 ab	420.0 ab
	200 mg L^−1^	4.40 ab	0.30 ab	506.67 ab
	400 mg L^−1^	4.40 ab	0.27 abc	436.0 ab
I4	0 mg L^−1^	3.26 bc	0.25 abcd	588.0 a
	200 mg L^−1^	4.57 a	0.28 abc	552.0 ab
	400 mg L^−1^	5.21 a	0.34 a	424.0 ab

I1: one time irrigation; I2: two times irrigation; I3: three times irrigation; I4: four times irrigation. Different letters (a–e) in columns indicate significant differences as tested by Tukey’s HSD test at >0.05.

**Table 7:** Grain relative yield reduction (RYD) of application treatments.

Silicate Rates	Grain Yield (kg ha^−1^)				RYD (%)		
	Irrigation Treatments						
	I1	I2	I3	I4	I1	I2	I3
0 mg L^−1^	950	2860	4480	3260	70.9	12.3	0.0
200 mg L^−1^	2400	2170	4400	4570	47.5	52.5	3.7
400 mg L^−1^	2980	2480	4400	5210	42.8	52.4	15.5
Average	2110.0	2503.3	4426.7	4346.7	53.7	39.1	6.4

I1: one time irrigation; I2: two times irrigation; I3: three times irrigation; I4: four times irrigation.

**Table 8:** Irrigation water productivity (IWP) concerning applied irrigation water (AIW) under applied irrigation and sodium silicate treatments.

Irrigation Treatments	Sodium Silicate	AIW (mm)	AIW (m^3^ ha^−1^)	Grain Yield (kg ha^−1^)	IWP (kg m^−3^)
I1	0 mg L^−1^	100	1000	950	0.95
	200 mg L^−1^	100	1000	2400	2.40
	400 mg L^−1^	100	1000	2980	2.98
	Average of two seasons	100	1000	2110	2.11
I2	0 mg L^−1^	200	2000	2860	1.43
	200 mg L^−1^	200	2000	2170	1.09
	400 mg L^−1^	200	2000	2480	1.24
	Average of two seasons	200	2000	2503	1.25
I3	0 mg L^−1^	300	3000	4480	1.493
	200 mg L^−1^	300	3000	4400	1.467
	400 mg L^−1^	300	3000	4400	1.467
	Average of two seasons	300	3000	4427	1.48
I4	0 mg L^−1^	400	4000	3260	0.815
	200 mg L^−1^	400	4000	4570	1.143
	400 mg L^−1^	400	4000	5210	1.303
	Average of two seasons	400	4000	4347	1.09

I1: one time irrigation; I2: two times irrigation; I3: three times irrigation; I4: four times irrigation.

**Table 9:** The recommended amounts of applied irrigation water (AIW).

Month	ETo (mm)	Kc	ETc (mm day^−1^)		AIW (mm day^−1^)	
			mm	m^3^ ha^−1^	mm	m^3^ ha^−1^
November	57.8	0.40	23.1	231	30.8	308
December	96.3	0.62	59.7	597	79.6	796
January	91.1	1.00	91.1	911	121.5	1215
February	84.1	0.85	71.5	715	95.4	954
March	141.8	0.83	117.7	1177	157.0	1570
April	65.6	0.20	13.1	131	17.5	175
Total	536.7		376.3	3763	501.7	5017

ETc (mm day^−1^) and applied irrigation water values are expressed in mm and m^3^ ha^−1^.

## Data Availability

The datasets used and/or analyzed during the current study are available from the corresponding author.
